# The utility of scores in the decision to salvage or amputation in severely injured limbs

**DOI:** 10.4103/0019-5413.43371

**Published:** 2008

**Authors:** Rajasekaran Shanmuganathan

**Affiliations:** Department of Orthopaedics and Spine Surgery, Ganga Hospital, Coimbatore 641043, India

**Keywords:** Open fractures, severely injured limbs, limb injury severity score

## Abstract

The decision to amputate or salvage a severely injured limb can be very challenging to the trauma surgeon. A misjudgment will result in either an unnecessary amputation of a valuable limb or a secondary amputation after failed salvage. Numerous scores have been proposed to provide guidelines to the treating surgeon, the notable of which are Mangled extremity severity score (MESS); the predictive salvage index (PSI); the Limb Salvage Index (LSI); the Nerve Injury, Ischemia, Soft tissue injury, Skeletal injury, Shock and Age of patient (NISSSA) score; and the Hannover fracture scale-97 (HFS-97). These scores have all been designed to evaluate limbs with combined orthopaedic and vascular injuries and have a poor sensitivity and specificity in evaluating IIIB injuries. Recently the Ganga Hospital Score (GHS) has been proposed which is specifically designed to evaluate a IIIB injury. Another notable feature of GHS is that it offers guidelines in the choice of the appropriate reconstruction protocol. The basis of the commonly used scores with their utility have been discussed in this paper.

## INTRODUCTION

**Figure 1:**
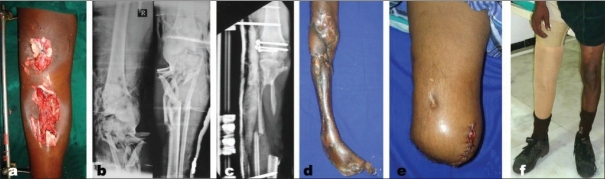
(a) Clinical photograph shows severe open injury of tibia with soft tissue loss. (b) X-rays (anteroposterior view) showing severe communition of the bones in proximal 1/3rd of tibia. (c) X-ray (anteroposterior view) showing gap non-union upper end tibia. (d) Clinical photograph of same patient showing an infected non-union with sinuses and a deformed foot. (e) Clinical photograph of same patient showing above-knee amputation. f) Clinical photograph after rehabilitation

Severe open injuries of limbs, especially of the tibia when associated with vascular injuries, present major challenges in management. The decision to amputate or salvage can often be a difficult one even for experienced surgeons.^1^–^3^ In the 1960s, the presence of a severe crush injury or a vascular injury was sufficient to warrant an amputation. However, the evolution of sophisticated microsurgical reconstruction techniques along with the development of modern skeletal fixation and reconstruction devices in the 1980s made limb salvage technically possible even in the most extreme cases. Surgeons began undertaking prolonged attempts at reconstruction, and patients who sustained severe Grade III B and C open tibia fractures were subjected to two to three years of hospitalization; multiple surgeries, sometimes up to 20 surgeries including debridement, fixation attempts, soft tissue cover procedures, and bone grafts, were performed.^3^ Despite such heroic but not very wise efforts, failures were common because of infection, nonunions, soft tissue cover failures, and delayed secondary amputation.^2^,^4^–^8^ In the process, many patients lost their jobs, families, savings, and most importantly, their self-image and self-respect^1^,^3^,^7^ As a result of secondary amputation, not just the limb is lost, but the patients and their families are frequently devastated and destroyed physically, psychologically, socially, and financially.^7^ It became obvious that technical advances can be double-edged swords, and prolonged attempts at salvage may actually be a “triumph of technique over reason” [Figure 1].

In attempting salvage, the question therefore is not “whether you can” but “whether you should or not.” There is good evidence that patients with primary amputation and who have been rehabilitated well not only perform better but are also saved of the agony of multiple surgical procedures and severe financial strain.^3^,^7^,^9^–^13^ However, a limb that could be saved must never be amputated. Open injuries are common in developing countries, where most amputees do not have the access to modern prosthetic devices. Thus, there is a need for objective and reliable methods of assessing a severely injured limb and for predicting a good outcome.^7^

## OBJECTIVE ASSESSMENT OF OPEN INJURIES

The decision to amputate a limb is chiefly mandated by the severity of injury to the lower limb, associated injuries, and the health status of the patient. However, the assessment of severity of injury to the limb is usually done based on subjective criteria rather than objective criteria. The fallacy of this method led several authors to attempt to quantify the severity of trauma and to propose scores so as to establish numerical guidelines. The currently available scores include the Mangled Extremity Severity Score (MESS),^14^ the Predictive Salvage Index (PSI);^15^ the Limb Salvage Index (LSI);^16^ the Nerve Injury, Ischemia, Soft Tissue Injury, Skeletal Injury, Shock, and Age of the Patient (NISSSA) score;^17^ the Hannover Fracture Scale-97 (HFS-97),^18^ and the Ganga Hospital Open Injury Severity Score (GHOISS)^19^ [Table 1].

**Table 1:** Variables in different limb injury severity scores

	With associated vascular injuries					IIIB injuries
		
	MESS	LSI	PSI	NISSSA	HFS-97	GHOISS
Age	x			x		x
Shock	x			x	x	x
Warm ischemia time	x	x	x	x	x	x
Bone injury		x	x		x	x
Muscle injury		x	x			x
Skin injury		x			x	x
Nerve injury		x		x	x	
Deep-vein injury		x				
Skeletal/soft tissue injury	x			x		
Contamination				x	x	x
Time to treatment		x			x	
Co-morbid conditions						x

MESS-Mangled Extremity Severity Score: LSI-Limb Salvage Index; PSI-Predictive Salvage Index: NISSSA-Nerve Injury, Ischemia, Soft tissue injury, Skeletal injury, Shock and Age of the patient; HFS-97-Hannover Fracture Scale; GHOISS-Ganga Hospital Open Injury Severity Score

## AN IDEAL LIMB SALVAGE SCORE

An ideal score must fulfill a few basic criteria before it can be accepted as a clinical guideline. The score must perform consistently and with a high degree of sensitivity and specificity not only in a small retrospective series but also in a larger population of patients when applied prospectively and in a multicenter trial.^11^,^20^ If it has to be practical and useful, it must be simple and readily applicable in the operating room. The number of variables must be less, and these variables must have a high interobserver agreement rate.^21^ The study cohort on which these variables have been validated must ideally have not grouped upper and lower limbs together because these two have different prognosis, need for salvage, and disability on amputation.

Ideally, a limb salvage score should be 100% sensitive (all amputated limbs will have trauma limb salvage scores at or above the threshold) and 100% specific (all salvaged limbs will have scores below the threshold). However, this level of accuracy is impossible in any clinical setting, especially in an open injury, where the variables influencing the outcome are often difficult to numerically quantify and not confined to the status of the limb or the even the individual. There are important external factors such as the technical facilities available and the surgical skills of the treating team.^2^,^21^ Hence, it is more practical to look for the highest possible rate of sensitivity and specificity rather than a 100% perfect accuracy. A high rate of specificity is more important so that we can significantly reduce the occurrence of salvageable limbs being incorrectly assigned to a score above the decision threshold and being unnecessarily amputated. However, sensitivity is also important so as to avoid inappropriate attempts at salvage with its associated high morbidity and even mortality.

## AVAILABLE CLASSIFICATIONS AND SCORES

### Gustilo–Anderson's classification

**Figure 2:**
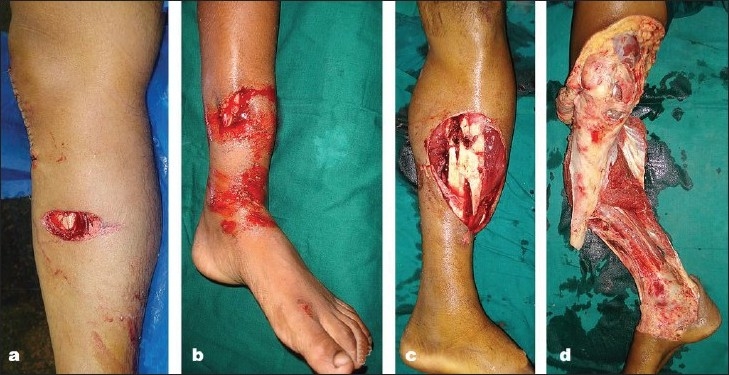
Clinical photograph of four different injuries, (a-d) which are all Gustilo IIIB by definition. Management and outcome of all these injuries, although grouped together under IIIB, are completely different

A major advance in our understanding of open injuries was achieved with Gustilo classification. It is the most widely used system, which established the correlation of the severity of injury to outcomes.^22^,^23^ Several studies have shown an increase in complication rates and poor results in Grade IIIB injuries when compared with those in less severe injuries. The amputation rates are very high in Grade IIIC injuries and can be from 59 to 90% depending on the associated factors and the availability of skilled microsurgical reconstruction facilities. However, Gustilo classification was primarily designed to indicate the need for soft tissue coverage, and there are several disadvantages in utilizing the classification for salvage. Different interpretations by various authors have resulted in loss of uniformity in its global understanding and application.^21^ Grade IIIB includes a wide spectrum of injuries from the easily manageable to the barely salvageable and is therefore unable to provide guidelines for management [Figure 2].^19^ The system also does not consider comorbid factors and does not address the question of salvage.^19^ There is a high degree of subjectivity leading to poor inter-observer reliability.^21^,^24^ Two major studies evaluating the Gustilo classification have reported a low interobserver agreement rate (60%), which varied with the experience of the surgeon and the type of injury.^21^,^24^

Many authors have suggested the need to have a more accurate and objective method of predicting salvage and outcomes, and this has led to the proposal of many different scores in the literature.^8^ Apart from the GHOISS, all the other scores are designed to evaluate the outcome in combined orthopedic and vascular injuries.

### Predictive salvage index

The predictive salvage index (PSI) was proposed by Howe *et al*.^15^ in the year 1987 to avoid protracted attempts at salvage, especially in patients who had a combination of limb injuries associated with vascular injuries [Table 2]. The main aim was to avoid an unnecessary or a delayed amputation of a limb. The study was based on a retrospective analysis of a small group of 21 limbs, which analyzed the variable factors that determined amputation or salvage in that group. The variables that were given importance were the extent of vascular injury, the degree of bone damage, the degree of injury to the muscles, and the warm ischemia time. Howe *et al*. reported a sensitivity of 78% and a specificity of 100% in their cohort of patients.

**Table 2:** Predictive salvage index

Artery	
Supra popliteal level	1
Popliteal level	2
Infra popliteal level	3
Bone	
Mild	1
Moderate	2
Severe	3
Muscle	
Mild	1
Moderate	2
Severe	3
Interval up to operating theatre	
<6 Hour	1
6–12 Hours	2
> 12 hours	3

### Mangled extremity severity score

The mangled extremity severity score (MESS) was reported in 1990 by Johansen *et al*.^14^ to assist in the decision of injuries that also had a vascular component [Table 3]. A strong weightage was given for the presence of warm ischemia time and an age above 30 years. As the “vascular injury” was not clearly defined, the MESS has been used extensively for the evaluation of limbs with normal vascularity also. The MESS evaluates four important variables: degree of injury to the tissues, presence and duration of shock, age of the patient, and the severity and duration of limb ischemia. The score was initially developed by a retrospective analysis of 25 patients and subsequently prospectively in a group of 26 limbs. Johansen *et al*. reported that a score of 7 or more predicted amputation with 100% accuracy.

**Table 3:** Mangled extremity severity score

Skeletal/Soft tissue group		
Low energy	Stab wounds, simple closed fractures, small caliber gun shot wounds	1
Medium energy	Open or multiple level fracture, dislocations, moderate crush injuries	2
High energy	Shotgun blast (close range) high velocity gunshot wounds	3
Massive crush	Logging, rail road, oil rig accidents	4
Shock group		
Normotensive hemodynamics	Blood pressure stable in field and operating room	0
Transiently hypotensive	Blood pressure unstable in field but responsive to intravenous fluids	1
Prolonged hypotensive	Systolic blood pressure<90 mm Hg in field and responsive to intravenous fluid only in operating room	2
Ischemia group		
None	Pulsatile limb without signs of ischemia	0
Mild	Diminished pulses without signs of ischemia	1
Moderate	No pulse by doppler, sluggish capillary refill, paresthesia, diminished Motor activity	2
Advanced	Pulseless, cool, paralysed and numb without capillary refill	3
Age group		
<30 years		0
30-50 years		1
>50 years		2

If ischemia time more than six hours, add 2 points

**Figure 3:**
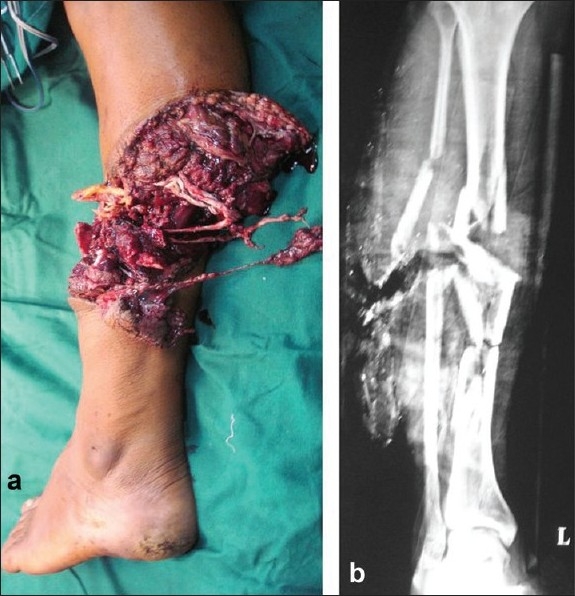
(a) Clinical photograph of leg with severe crushing of soft tissues with absence of a vascular injury. (b) X-ray of left leg bones (AP view) was showing comminuted fracture tibia with bone loss. MESS has poor senstivity for amputation. Attempted salvage of this leg wound have led to prolonged surgeries and probably a secondary amputation. In contrast, Ganga Hospital Score was 17 indicating the need for amputation. In IIIB injuries, Ganga Hospital Score was more sensitive than a MESS in predicting amputation

Few others also reported a good accuracy of the MESS.^25^–^28^ However, the MESS has two disadvantages. First, it assumes that the outcome in patients whose age is below 30 years and in the group whose age is between 30 and 50 years would be different. Although an age of above 50 years may affect the outcome, it is doubtful whether the outcome would be different between the two groups of age less than 30 years and 30–50 years. Second, it also assumes that even a temporary depression in the blood pressure at the time of presentation to the hospital could negatively affect the outcome of the patient. In our experience, the MESS is very useful in predicting limbs that could be salvaged but is less accurate in predicting limbs that require amputation when applied for the evaluation of severe IIIB injuries.^19^ In other words, it has good specificity but poor sensitivity for amputation. It is difficult to obtain a score of 7 and above when the vascularity is intact even though the bone and soft tissue damage is so extensive that salvage is impossible or doomed to fail. As a result, higher rates of limbs undergo failed attempts at salvage and secondary amputations. A typical example is shown in Figure 3.

### NISSSA score

In 1994, McNamara *et al*.^17^ proposed the NISSSA score, which had the addition of a nerve injury component [Table 4]. They divided the tissue injury into bony and soft tissue components and added a nerve injury component, giving the highest weight to the loss of plantar sensation. They thought that this was an improvement to the MESS, because they thought that amputation of a limb with complete loss of plantar sensation was inappropriate as it would ultimately lead to a useless limb. This was again a score proposed in retrospection of 26 limbs that were assessed both by MESS and by NISSSA. Both scores were found to be highly predictive of amputation, but the NISSSA was found to fair better compared with MESS in both sensitivity (81.8% *vs* 63.6%) and specificity (92.3% *vs* 69.2%).

**Table 4:** Nerve injury, ischemia, soft tissue injury, skeletal injury shock & age of patient score

Nerve		
Sensate	No major nerve injury	1
Dorsal	Deep peroneal nerve	2
Plantar partial	Tibial nerve injury	3
Plantar complete	Sciatic nerve	4
Ischemia		
None	Good to fair pulses, no ischemia	0
Mild	Decreased pulses perfusion	1
Moderate	Prolonged capillary refill, Doppler pulses fill	2
Severe	Pulseless, cool, ischemic, no doppler	3
Soft Tissue		
Grade I	Minimal contamination	0
Grade II	Moderate soft tissue injury, low velocity	1
Grade IIIA	Moderate crush injury, high velocity with Considerable contamination	2
Grade IIIB	Massive crush injury severe contamination	3
Skeletal		
Spiral or oblique fracture		0
Transverse fracture-minimal contamination		1
Moderate displacement and communition with high velocity		2
Segmental fracture, severe communition, bony loss		3
Shock		
Normotensive		0
Transient hypotensive		1
Persistent hypotensive		2

If ischemia time more than six hours, add 2 points

The NISSSA score has been found to be more accurate than MESS.^29^ However, the idea of placing too much weightage on loss of plantar sensation at presentation^19^ or even later^30^ has been criticized. Severe crush injuries of limbs may have an associated crush neuropraxia or minor avulsion injury of the posterior tibial nerve, which will improve spontaneously. In acute traumatic conditions, it may often be impossible to differentiate recoverable or permanent damage to the nerve. A false assessment of the plantar sensation loss may result in unnecessary amputation. It has been argued that a very functional limb can be obtained with proper rehabilitation and appropriate footwear even in the presence of a complete irreparable damage to the posterior tibial nerve.^19^

### Limb salvage index

The limb salvage index (LSI) was proposed by Russell *et al*.^16^ in 1991 again to assist in the evaluation of a limb with combined orthopedic and vascular injury [Table 5]. Seventy limbs were evaluated retrospectively, of which 26 had vascular injury requiring revascularization and a threshold score of 6 was proposed for amputation. The 7 variables regarding the injury were arterial, nerve, skeletal, skin, muscle, deep venous injury, and warm ischemia time. Vascular injury was divided into the two components of arterial and deep venous injury. Although not utilized widely, the score was found to fair better than the MESS, PSI, NISSSA, and HFS-97 when assessing Type III tibial fractures.^8^

**Table 5:** Limb salvage index

Artery	
Contusion, intimal tear, partial laceration	0
Occlusion of two or more shank vessels, no pedal pulses felt	1
Complete occlusion of femoral or three shank vessels	2
Nerve	
Contusions, stretch, minimal laceration	0
Partial transaction or avulsion of sciatic nerve	1
Complete transaction or avulsion of sciatic nerve	2
Bone	
Closed or open fracture with minimum communition	0
Closed fracture at two or more sites at same limb;	1
Open fracture with communition or moderate to large displacement with bone loss < 5 cm	
Bone loss more than 5 cm Grade IIIB or IIIC	2
Skin	
Clean injury, primary repair, first degree burn	0
Delayed closure due to contamination requiring skin graft or flap, second or third degree burn	1
Musculotendinous unit	
Laceration or avulsion involving the single compartment or tendon	0
Complete avulsion injury involving two or more tendon	1
Deep Vein	
Contusion, partial laceration	0
Complete laceration, avulsion or thrombosis	1
Warm ischemia	
Less than six hours	0
6-9 hours	1
9-12 hours	2
12-15 hours	3
More than 15 hours	4

### Hannover fracture scale

Thirteen characteristics related to severity of injury were weighted to give a hannover fracture scale (HFS) reported initially in 1993.^18^ The variables and their respective weightage given to each have been modified later because of refinement by continued reassessment strategy with the use of multiple regression analysis and receiver operator characteristic curves. This score is also heavily biased toward the presence of vascular injuries and is meant to assess injuries with orthopedic and vascular injuries. Apart from being cumbersome, the need for advanced bacteriological studies of specimens from the initial wound has prevented the wide usage of the score. The nonavailability of this facility makes the score inapplicable in many centers and also makes it impossible to complete during the index procedure.

### Assessment of severe open injuries without vascular deficit

All the abovementioned scores were proposed mainly for the assessment of combined vascular and orthopedic injuries. A high weightage for vascular injuries has been built into the scores, and all of them perform poorly in the absence of a vascular injury even though the limb may be severely injured and beyond salvage. Although the danger of limb loss is well recognized in Grade IIIC injuries, there can be frequent management dilemmas in Grade IIIB injuries also and errors of inappropriate limb salvage are frequent in Grade IIIB injuries because of the lack of appropriate guidelines.

The Ganga hospital open injury severity score (GHOISS) is unique, in that it has been evolved to address the need of assessment of severe Grade IIIB injuries.^19^

### Ganga Hospital Open Injury Severity Score

**Figure 4:**
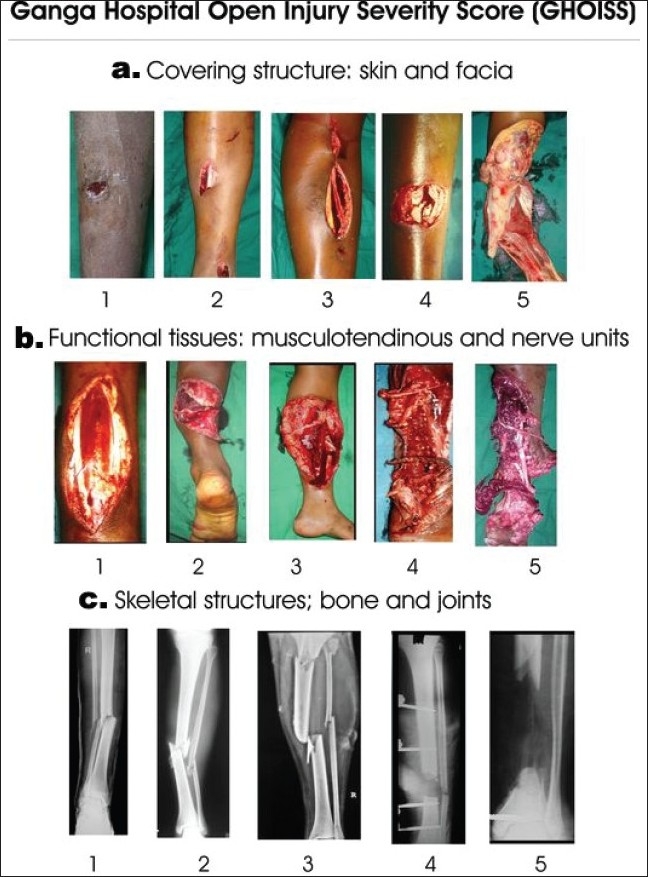
The various components in the respective scores of the Ganga Hospital Score

GHOISS was proposed by Rajasekaran *et al*. in 2006^19^ as a score specifically to assess severe Grade IIIB limb injuries without a vascular injury [Table 6]. The score was developed in 1994 and was subsequently modified to the published form after three clinical trials. It assessed the severity of the injury to the limb separately to each of the three components of the limb: the covering tissues (skin and facia), the skeleton (bones and joints), and the functional tissues (muscles, tendons and nerve units) [Figure 4]. Seven systemic factors, which may influence, the treatment, and outcome were given two points each, and the final score is arrived by adding all the individual scores together. The total score was used to assess the possibilities of salvage, and the outcome was measured by dividing the injuries into four groups according to their scores as follows: group 1 scored less than 5, group II 6–10, group III 11–15, and group IV 16 or more. The score was validated in 109 consecutive open injuries of the tibia (42 Grade IIIA and 67 Grade IIIB injuries).

**Table 6:** Ganga Hospital Open Injury Severity Score

Covering structures: Skin and fascia	Score
Wounds with out skin loss	
Not over the fracture	1
Exposing the fracture	2
Wounds with skin loss	
Not over the fracture	3
Over the fracture	4
Circumferential wound with skin loss	5
Skeletal structures: Bone and joints	
Transverse/oblique fracture/butterfly fragment < 50% circumference	1
Large butterfly fragment > 50% circumference	2
Comminution/segmental fractures without bone loss	3
Bone loss < 4 cm	4
Bone loss > 4 cm	5
Functional tissues: Musculotendinous (MT) & Nerve units	
Partial injury to MT unit	1
Complete but repairable injury to MT units	2
Irreparable injury to MT units/partial loss of a compartment/complete injury to posterior tibial nerve	3
Loss of one compartment of MT units	4
Loss of two or more compartments/subtotal amputation	5
Co-morbid conditions: Add 2 points for each condition present	
Injury – debridement interval > 12 hrs Sewage or organic contamination/farmyard injuries Age > 65 yrs Drug dependent diabetes mellitus/cardio respiratory diseases leading to increased anesthetic risk Poly trauma involving chest or abdomen with ISS>25/Fat embolism. Hypotension with systolic blood pressure<90mm Hg at presentation. Another major injury to the same limb/compartment syndrome	

A score of 14 to indicate amputation had the highest sensitivity and specificity. GHOISS was found to compare favorably with the MESS in sensitivity (98% *vs* 99%), specificity (100% *vs* 17%), positive predictive value (100% *vs* 97.5%), and negative predictive value (70% *vs* 50%). The scoring system was found to be simple in application and reliable in prognosis for salvage and outcome measures.

A unique factor of the score was that it provided thresholds for salvage and amputation and also a grey zone in between. Rajasekaran *et al*. emphasized that injuries with a score of 14 and below should be attempted for salvage, those with the score of 17 and above should be considered for primary amputation, and those in between must be assessed by an experienced team on a case-to-case basis. They stated that it was important to have an intermediate grey zone rather than a strict threshold score because the management of these severe injuries is influenced by many other factors such as skill and experience of the treating team, the social and cultural background of the patient, the cost, and the personality of the patient.

**Figure 5:**
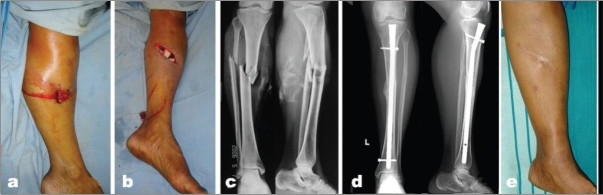
(a & b) Clinical photograph showing open injury of the tibia with exposed bone. (c) X-ray anteroposterior and lateral views of leg bones showing comminuted fracture of tibia. As per the Ganga Hospital Score, the total score is less than 5 and the skin score is less than 3. (d) X-ray anteroposterior and lateral view of leg bones showing skeletal fixation and union. (e) Clinical photograph showing result after immediate skin closure and a thorough debridement leading to good result

**Figure 6:**
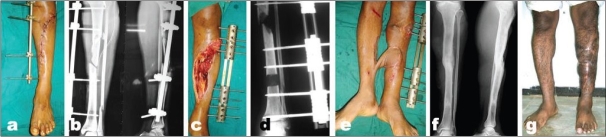
(a) Clinical photograph shows severe open injury of tibia with Ganga Hospital score more than 10. A score above 10 indicates a high velocity injury and primary reconstruction will not be successful. (b) Radiograph (anteroposterior and lateral) shows temporary stabilization. (c) Clinical photograph after debridement. (d) Radiograph (anteroposterior) shows bone transport procedure. (e) Clinical photograph shows cross leg flap. (f) Radiograph (anteroposterior and lateral) shows union at final follow up. (g) Clinical photograph shows weight bearing extremity with limb length equality

GHOISS, apart from proposing boundaries for amputation and salvage, also allowed guidelines for reconstruction protocols.^31^ Most of the failures of open injury management lie in inappropriate timing or sequence of reconstruction procedures.^31^ An analysis of 728 Grade III open fractures showed that primary closure policy in open fractures is safe whenever the total score is less than 5 or the skin score is 1 or 2 [Figure 5]. Similarly, early flaps were safe when the skin score was 3 and above but the total score was less than 10. A total score of less than 10 denoted low-velocity injuries, and it allowed early soft tissue reconstruction [Figure 6]. However, when the total score exceeded 10, the injuries were usually high-velocity injuries with an extensive zone of injury. Here, it was more appropriate to allow time to let the edema settle and then perform the reconstruction.

## CRITICISM OF SCORES

The validity of usage of scores for assessing salvage has been questioned by the Lower Extremity Injury Severity Scores (LEAP) study. LEAP was a prospective longitudinal study of 601 patients with a severely injured lower limb and included in the study with strict inclusion and exclusion criteria.^7^ Patients were admitted to one of the eight Level 1 trauma centers in the United States for the treatment of high-energy trauma of the lower extremity. As a part of the major study, the clinical utility of five lower extremity injury severity scoring systems (MESS, LSI, PSI, NISSSA, and HFS-97) in predicting amputation were analyzed. In the final analysis, the authors reported that their study could not validate the clinical utility of any of the abovementioned lower extremity injury severity scores. They concluded that the scores were quite useful in predicting limb salvage, but the opposite (i.e., decision to amputate) was not true. All the scores in the series had low sensitivity and could not be accurate predictors of amputation.

Although the LEAP study is the first large prospective study on this difficult subject, it should be remembered that all participating centers were restricted to the United States, and hence, its results cannot be extrapolated universally. The acceptance of amputation and the social stigma and psychological impact of amputation are quite different in various societies and geographical regions of the globe. The cost provider and social support to help the patient through the various reconstructive surgical procedures and the rehabilitation process after amputation or salvage are totally different in different countries, and it is incorrect to propose universal guidelines of management based on the sickness impact profile (SIP) measured at two years in American patients. Although prospective, it was still not a randomized trial, and the decision to amputate or salvage was based on the operating surgeon's judgment from the various centers and hence understandably may not have been uniform. The patients were included prospectively, but many of the analysis have been performed retrospectively.

LEAP study also indicated that at two years, functional outcomes after amputation were similar to those after reconstruction. The differences between the treatment groups were not significant in the proportion of patients who were returned to work at two years. To accept that results of salvage and amputation of the same is to assume that all patients will have access to state-of-the art prosthesis devices that are available in the West. Amputated patients need to have a new prosthesis every two or three years at an average cost of US$7784 each for below knee prosthesis, US$16 028 for a through-the-knee prosthesis and US$18 722 for an above-knee prosthesis. Such economic scales are unrealistic in many parts of the world and almost always are unavailable to patients in developing countries. Poor quality prosthesis will obviously decrease the patient's satisfaction and outcome results following amputation. The stigma of amputation, life conditions of the rural population, the accessibility, and affordability to state-of-the art prosthesis in patients in developing countries are entirely different to the United States, and the threshold for amputation must naturally be higher in these countries. The practice of medicine in the West is increasingly dictated by the insurance companies, and fortunately this is not the situation in the East. Hence, although the LEAP study is a great step forward in our analysis and understanding of the outcomes following lower limb trauma, we should be wary of extending their conclusions to patients in India and other developing countries. There is an urgent need for similar studies in the developing countries.

## ARE SCORES INFALLIBLE?

The utility of scoring systems in many fields of medicine has been validated for classification of severity of diseases, quality assurance, providing management guidelines, comparison of results from different institutions, and cost performance among patient groups. Although they are not infallible, they are widely used by physicians while facing a challenging medical decision. Scores often work on a threshold value for a treatment decision. This has been criticized especially in crucial decisions such as amputation. These criticisms should not be taken as a pitfall of the scores but should rather be seen in proper perspective. The success of the treatment in open injuries depend on not only the severity of limb injury but also a variety of factors such as associated injuries and comorbid factors of the patient, the facilities available and the expertise of the treating team. What is salvageable in an advanced center may not be so in another less-equipped center. It is obvious that the threshold value for salvage may differ from center to center, but each team should endeavor to identify the particular score and the threshold value, which is applicable to them.

In conclusion, scores are infallible and scores are not useful are two extreme points of view, which are both not true. In a doubtful situation, scores do provide additional guidelines in the management of a problem. They also allow comparison of patient cohorts from different institutions and countries and allow evaluation and comparison of different treatment regimes for the same severity of injury. There is however no doubt that the surgeon should ultimately decide on each case based on the severity of injury, the health status of the patient, the decision of the informed patient and his family, the level of technical facilities available, and last but not the least, his/her own personal skill and experience.
